# The management of diabetic ketoacidosis at a rural regional hospital in KwaZulu-Natal

**DOI:** 10.4102/phcfm.v10i1.1612

**Published:** 2018-03-22

**Authors:** Nontobeko F.M. Ndebele, Mergan Naidoo

**Affiliations:** 1 School of Clinical Medicine, University of KwaZulu-Natal, South Africa; 2 School of Nursing and Public Health, University of KwaZulu-Natal, South Africa

## Abstract

**Background:**

Diabetic ketoacidosis (DKA) is a biochemical triad of hyperglycaemia, ketoacidosis and ketonaemia and one of the potentially life-threatening acute metabolic complications of diabetes mellitus. This study aimed at describing the clinical profile of patients presenting with DKA to a busy rural regional hospital in KwaZulu-Natal.

**Methods:**

A retrospective review of clinical notes of patients presenting with DKA to the Emergency Department was performed over a 10-month period. Data included patients’ demographic profile, clinical presentation, precipitating factors, comorbidities, biochemical profile, length of hospital stay and outcome.

**Results:**

One hundred and five black South African patients above the age of 12 years were included in the study. Sixty-four (60.95%) patients had type 1 diabetes mellitus (T1DM) and 41 (39.05%) patients had type 2 diabetes mellitus (T2DM). Patients with T2DM were significantly older than those with T1DM (52.1 ± 12.4 years vs. 24.4 ± 9.5 years, p < 0.0001). The acute precipitant was identified in 68 (64.76%) cases with the commonest precipitant in T1DM patients being poor adherence to treatment, whereas in T2DM, the most common precipitant was infection. Nausea and vomiting were the most common presenting symptoms with the majority of patients presenting with non-specific symptoms. Fifty-seven (54.29%) cases had pre-existing comorbidities, with higher prevalence in T2DM than T1DM patients. Glycated haemoglobin was severely elevated in the majority of patients. Patients remained hospitalised for an average of 8.9 ± 7.5 days. The mortality rate was 17.14%, and 12 of the 18 deaths occurred in patients with T2DM.

**Conclusion:**

The prevalence of DKA was higher in patients with T1DM and those with pre-existing comorbidities. The mortality rate remains alarmingly high in older patients with T2DM.

## Introduction

Diabetic ketoacidosis (DKA) is a potentially life-threatening emergency condition caused by acute hyperglycaemia that may be associated with both type 1 and type 2 diabetes mellitus.^1^ The cardinal biochemical manifestations include hyperglycaemia, ketonaemia or ketonuria and metabolic acidosis.^2^ Normoglycaemic DKA has also been described.^3^ Classically, patients present with clinical features that may include a history of polyuria, polydipsia, polyphagia, nausea and vomiting.^4^ But, over the last decade, there has been a change in the way patients with DKA present^5^ as more patients tend to now present with a variety of non-specific symptoms that may result in the diagnosis being missed by the primary care physician.^6,7^

Infection and poor compliance to treatment are the leading triggers in the development of DKA.^8,9,10^ Patients with undiagnosed diabetes mellitus may present primarily with DKA to their health care provider.^6,11^ There are instances where no precipitating factors could be identified.^12^ DKA tends to be associated with elevated HbA1c levels in all patients whether they are known with diabetes or newly diagnosed.^13^ Comorbidities play a major role in the outcome of patients that present with DKA.^14^ Mortality rates generally remain high, but there is significant variation between high- and low- and middle-income countries.^1^ Because of resource constraints, mortality rates also show great variation in low- and high-income countries (HICs).^15,16^ Contributing to mortality rates are the delays in diagnosing and treating this acute medical emergency.^17^

The diagnosis of DKA is made based on consensus statement by the American Diabetes Association which recommended the triad of glucose more than 13.9 mmol/L, pH less than 7.35 and the presence of ketonaemia and/or ketonuria.^18^ Severity of DKA can be classified by measuring arterial blood pH. Intravenous replacement of fluid deficit and correction of ketoacidosis with fixed rate insulin infusions remain the cornerstone of DKA treatment.^12^ In this study, the standard DKA management protocol that is currently being used in the Emergency Department (ED) was followed to treat all patients that were diagnosed with DKA. All patients with DKA were first admitted to Resuscitation Unit (RU) in the ED for stabilisation before being transferred to speciality units. Beyond acute management, it is essential to implement a comprehensive approach for appropriate long-term treatment of diabetes, in order to prevent recurrence of DKA.^7^

This study aimed at describing the clinical profile, precipitating factors and outcomes of patients presenting with DKA at Ngwelezana Regional Hospital in KwaZulu-Natal (KZN). The hospital is the only regional referral hospital in Northern KZN and serves 18 district hospitals from three health districts with an estimated catchment population of 2.3 million people. There are few other studies both locally and internationally that have described the profile of DKA patients from such a setting.^8,19,20^

## Methods

A retrospective review of clinical files from patients presenting to the ED at Ngwelezana Hospital was performed. Clinical files from patients above the age of 12 years in which the diagnosis of DKA had been made were included in the study. Only patients who presented between March 2015 and December 2015 were considered for inclusion. Patients were classified as type 1 diabetes mellitus (T1DM) or type 2 diabetes mellitus (T2DM) based on the history obtained at presentation. In addition, clues from their treatment regimens suggested their type, that is, those with the requisite phenotypical characteristics on injectable insulin only were assumed to have T1DM and those with certain phenotypical characteristics on oral hypoglycaemic agents or both oral medication and injectables were assumed to have T2DM. Clinicians working in the ED asked patients about medication compliance routinely and this was documented in the clinical files.

Data from the medical records were collected using a data collection tool, which included variables such as the demographic profile, the clinical presentation, diabetes type, precipitating factors, existing comorbid condition(s), serum biochemical measurements at presentation, length of stay in hospital and the patient’s outcome. Patients presenting with DKA were actively screened by emergency medicine staff for infection and obvious sources of infection such as a septic limb were documented in the clinical files. Investigations such as the C-reactive protein levels, white cell count and suggestive finding on urine dipstick and chest X-rays confirmed clinical suspicion of infection.

The data were captured on an Excel spread sheet and analysed with the aid of a biostatistician from the University of KwaZulu-Natal (UKZN). Data were presented as means with standard deviations and medians with ranges for continuous variables and proportions for discrete variables. A comparison was made between the presentation and outcomes of patients with T1DM with those of patients with T2DM.

Differences between groups were tested statistically using the chi-squared test and independent samples t-test. Logistic regression models were fitted to compare risk of mortality. Data were analysed using SPSS version 23. Differences were considered statistically significant at a p < 0.05.

### Ethical considerations

The confidentiality of patients was protected by de-identifying patients using participant numbers, and data collection sheets were stored in a lockable cupboard. Ethical approval was obtained from the University of KwaZulu-Natal Biomedical Research Ethics Committee (BREC) – BE 245/15, and gatekeeper permission was obtained from the hospital manager and the Provincial Department of Health.

## Results

Files of 105 participants with the diagnosis of DKA were reviewed. The age of the participants ranged from 14 to 75 years, with mean and standard deviation (SD) being 38.45 ± 16.6 years, respectively. Median age was 37 years and the male to female ratio was 1.05: 1. Table 1 cross-tabulates gender and age with mortality.

**TABLE 1:** Age and gender cross-tabulated with mortality.

Variables	Total (*N* = 105) *n* (%)	Discharges (*N* = 87) *n* (%)	Deaths (*N* = 18) *n* (%)
**Gender**			
Male	54 (51.4)	45 (51.7)	9 (50.0)
Female	51 (48.6)	42 (48.3)	9 (50.0)
**Age in years**			
< 20	14 (13.3)	13 (14.9)	1 (5.6)
21–30	27 (25.7)	26 (29.9)	1 (5.6)
31–40	20 (19.1)	16 (18.4)	4 (22.2)
41–50	14 (13.3)	10 (11.5)	4 (22.2)
51–60	20 (19.1)	14 (16.1)	6 (33.3)
61–70	7 (6.7)	5 (5.8)	2 (11.1)
71–80	3 (2.9)	3 (3.5)	0 (0.0)

Percentage of total cases in brackets.

There were no significant differences in age between male and female (35.6 ±14.3 vs. 41.4 ± 18.3 years) with most of the participants (25.7%) being in the 21–30 age group. The majority (60.95%) of the patients were classified as having T1DM, with a mean age of 24.4 ± 9.5 years. Twenty-seven (25.71%) of these patients were newly diagnosed patients with T1DM. Forty-one (39.05%) patients had T2DM, with a mean age of 52.1 ± 12.4 years. Patients with T2DM were significantly older than those with T1DM (p < 0.0001). The clinical presentation and identified precipitants as well as baseline comorbidities are presented in the two subgroups in Table 2.

**TABLE 2:** Precipitants, presenting symptoms and comorbidities cross-tabulated in both subgroups.

Measured variables	T1DM *n* (%)	T2DM *n* (%)	Frequency *n* (%)	*p*
**Precipitants identified**				
No precipitant documented	33 (31.43)	4 (3.81)	37 (35.24)	< 0.05
Infection or sepsis	7 (6.67)	22 (20.95)	29 (27.62)	0.03
Poor compliance	20 (19.05)	8 (7.62)	28 (26.67)	0.11
Infection and poor compliance	1 (0.95)	3 (2.86)	4 (3.81)	0.46
Other	3 (2.86)	4 (3.81)	7 (6.67)	0.35
**Presenting symptom**				
Lethargy and vomiting	19 (18.10)	14 (13.33)	33 (31.43)	0.52
Lethargy	4 (3.81)	8 (7.62)	12 (11.43)	0.39
Lethargy, vomiting and abdominal pain	6 (5.71)	4 (3.81)	10 (9.52)	0.66
Reduced level of consciousness	6 (5.71)	5 (4.76)	11 (10.48)	0.84
Septic limb	2 (1.90)	4 (3.81)	6 (5.71)	0.55
Vomiting	4 (3.81)	2 (1.91)	6 (5.71)	0.58
Shortness of breath	5 (4.76)	0 (0.00)	5 (4.76)	0.15
Polyuria, polydipsia and polyphagia	4 (3.81)	1 (0.95)	5 (4.76)	0.38
Shortness of breath and lethargy	3 (2.86)	2 (1.91)	5 (4.76)	0.76
Vomiting and abdominal pains	4 (3.81)	0 (0.00)	4 (3.81)	0.21
Polyuria, polydipsia, polyphagia and lethargy	4 (3.81)	0 (0.00)	4 (3.81)	0.21
Other	3 (2.86)	1 (0.95)	4 (3.81)	0.51
**Comorbidities**				
No comorbidity	38 (36.19)	10 (9.52)	48 (45.71)	< 0.05
Hypertension	8 (7.62)	18 (17.14)	26 (24.76)	0.14
HIV	6 (5.71)	4 (3.81)	10 (9.52)	0.66
HIV and PTB	3 (2.86)	1 (0.95)	4 (3.81)	0.51
HIV and hypertension	0 (0.00)	4 (3.81)	4 (3.81)	0.12
Hypertension and epilepsy	2 (1.90)	2 (1.90)	4 (3.81)	1.00
Other	7 (6.67)	2 (1.90)	9 (8.57)	0.27

Percentage of total cases in brackets.

PTB, pulmonary tuberculosis; T1DM, type 1 diabetes mellitus; T2DM, type 1 diabetes mellitus.

Precipitating factors were identified in 64.76% of cases, with infection and poor compliance being the commonest precipitants. The odds of a patient with T2DM compared to a patient with T1DM presenting with DKA that was precipitated by infection was 11.13 (95% CI: 3.86; 33.49), which is statistically significant. Lethargy and vomiting was the most commonly occurring presentation accounting for 31.43% of cases. Hypertension was the commonest comorbidity seen and present in 26 (24.76%) cases as a single comorbidity or in combination with other comorbidities (7.62%). Twenty-four (22.56%) out of the 34 participants had hypertension as well as T2DM. Table 3 provides a summary of the laboratory results for the participants at the time of presentation in both subgroups.

**TABLE 3:** Summary of laboratory investigations at presentation showing mean and standard deviation.

Variable	Normal values	T1DM	T2DM	*p*
Arterial pH	7.35–7.45	7.01 ± 0.1	7.1 ± 0.1	0.06
HCO3 (mmol/L)	18–28	8.9 ± 4.9	11.6 ± 5.5	0.01
Sodium (mmol/L)	135–147	134.3 ± 7.6	131.5 ± 7.4	0.06
Potassium (mmol/L)	3.3–5.3	4.9 ± 1.2	4.8 ± 0.8	0.63
Urea (mmol/L)	2.6–7.0	9.9 ± 8.7	12.8 ±14.2	0.24
Creatinine (µmol/L)	60–120	172.3 ± 117.5	174.3 ± 178.1	0.97
White cell count (× 10^9^/L)	4–10	16.2 ± 8.2	15.6 ± 9.2	0.72
Haemoglobin (g/dL)	14.3–18.3	13.4 ± 2.4	11.9 ± 2.7	< 0.01
Platelets 10^9^/L	135–373	338.4 ± 161.8	319.4 ±151.7	0.54
CRP (mg/L)	0–10	60.2 ± 103.3	212.8 ± 188.5	< 0.01
ESR (mm)	0–10 in 1 h	33.6 ± 36.3	68.3 ± 50.4	< 0.01
Lactate (mmol/L)	0.6–2.45	2.1 ± 1.6	2.1 ± 1.4	0.92
Amylase (IU/L)	28–110	137.7 ± 203.9	114.2 ± 157.8	0.56
Lipase (IU/L)	0–160	55.1 ±68.3	117.4 ± 356.7	0.23
HbA1C (%)	< 7	14.2 ± 2.3	14.6 ± 3.1	0.45
Blood glucose (mmol/L)	4.1–11.1	24.7 ±7.1	25.8 ± 4.2	0.40
Ketones (mmol/L)	0–2	2.91 ±0.4	2.51 ± 0.7	< 0.05

Normal measurement indices in brackets.

HCO_3_, serum bicarbonate; CRP, C-reactive protein; ESR, erythrocyte sedimentation rate; HbA1c, glycated haemoglobin; HGT, haemoglucotest; T1DM, type 1 diabetes mellitus; T2DM, type 1 diabetes mellitus.

There were significant differences in the serum concentration of HCO_3_, haemoglobin, CRP, ESR, ketones and HGT in both subgroups, with the haemoglobin and ketones being significantly higher in the T1DM group.

The mean number of days in the RU was 1.51 (± 0.8) days. Patients remained hospitalised for an average of 8.9 (± 7.5) days. Out of the 105 patients in the study, 18 died, resulting in a mortality rate of 17.14%. Twelve of the 18 deaths occurred in patients with T2DM. The mean age of those who died was significantly higher compared to those who were eventually discharged (47.2 ± 12.8 vs. 36.6 ± 16.8 years; p < 0.01).

Participants were categorised according to severity of DKA based on the arterial blood pH; this was cross-tabulated with outcomes and the results are outlined in Table 4.

**TABLE 4:** Diabetes type, diabetic ketoacidosis severity and the outcome.

Variables	Severity of diabetic ketoacidosis		
	Mild (*N* = 20) *n* (%)	Moderate (*N* = 59) *n* (%)	Severe (*N* = 26) *n* (%)
T1DM	6 (5.71)	40 (38.10)	18 (17.14)
T2DM	14 (13.33)	19 (18.10)	8 (7.62)
Deaths	4 (3.81)	8 (7.62)	6 (5.71)
Discharges	16 (15.24)	51 (48.57)	20 (19.05)

Percentage of total cases in brackets.

T1DM, type 1 diabetes mellitus; T2DM, type 1 diabetes mellitus.

Twenty patients (19.05%) had mild DKA, 59 patients (56.19%) had moderate DKA and 26 patients (24.76%) had severe DKA. Six deaths occurred in severe group, eight deaths in moderate group and four deaths in mild group. All patients with T1DM were receiving insulin only, whereas those with T2DM were being treated either with oral hypoglycaemic agents only or a combination of hypoglycaemic agent and insulin. The odds of dying from DKA were significantly lower in patients with T1DM than T2DM (OR = 0.32 [95% CI: 0.13; 0.79]).

## Discussion

Diabetic ketoacidosis occurred in patients with both T1DM and T2DM, but was more common in the former group. High prevalence of DKA in T1DM is a well-documented finding.^9,21^ Although it could manifest at any age, it is more common in the younger population. In this study, just over two-thirds of the participants were less than 50 years of age, which is in line with most of studies from HICs where the majority of patients with DKA were between the ages of 18 and 44 years.^18^

Patients with T2DM were significantly older than those with T1DM (p < 0.0001) and had more underlying comorbidities, which is consistent with other studies which found that such patients were often middle aged, obese and had other comorbidities.^19,22,23^ These patients also had a significantly higher mortality. Gender did not seem to have any effect on the frequency of DKA, which is consistent with what has been reported in the literature,^24,25^ but is at variance with other studies.^20,26,27^

Precipitating events associated with the development of DKA were identified in the majority of the participants, with the commonest being infection and treatment non-compliance. These leading triggers have also been reported in several other studies.^7,8,9,10,28^ Many studies showed that non-adherence alone was the main precipitant.^29,30,31,32,34^ Infections were more common in patients with T2DM, whereas poor compliance was the main precipitant in the T1DM group. The majority of patients with T1DM were young with poor compliance as the main precipitant which may have been because of peer influences, poor family involvement, high-risk behaviour, experimental behaviour, drugs and alcohol, and denial of the disease.^35,36^ The cause for poor compliance was not investigated. In patients with T2DM, higher occurrence of limb sepsis may have been associated with established neuropathy or vascular disease in an older population. Microvascular angiopathy has been described as a likely cause of limb sepsis in such patients.^37^ First presentation of diabetes mellitus as DKA is well documented and this occurred in 27 patients (25.71%) which is consistent with the literature.^6,11^ In some instances, no identifiable trigger could be found and this has also been well described.^34,38,39^

The majority of patients presented with lethargy and vomiting, but many of them had non-specific symptoms.^6^ This is expected as DKA presentation usually varies depending on severity, underlying precipitant(s) and comorbid conditions.^11^ The underlying physiologic disturbance also contributes to the presenting clinical picture as patients presenting with symptoms of polyuria, polyphagia, polydipsia and weight loss are likely to have hyperglycaemia, whereas gastrointestinal symptoms are more likely related to the degree of ketonaemia.^4^ Gastrointestinal manifestations could be the main findings in at least 50%–75% of patients and when misdiagnosed could lead to inappropriate management and catastrophic outcomes.^40^ The non-specific nature of the clinical presentation of DKA poses a challenge to the clinicians in recognising, diagnosing and instituting early treatment which predisposes to increased morbidity and mortality.^7^

In practice, DKA does not have any diagnostic symptoms that are sensitive or specific.^41^ This is despite the described classical clinical picture of polydipsia, polyuria, change in mental status, loss of weight, lethargy, vomiting and weakness, as these manifestations are not entirely unique to DKA patients and can also be present in patients with other metabolic conditions including the hyperosmolar hyperglycaemic state.^17^

Pre-existing comorbidities were identified in majority of T2DM patients who were significantly older compared to T1DM patients. This could be because elderly people generally have more underlying medical conditions by virtue of their age. Population-based studies have also shown that the prevalence of comorbidities together with the number of comorbid conditions increases with age.^14,42,43^

The mean duration of hospital stay was longer than the average length of stay for DKA patients in the United States which was 3.6 days^44^ and also longer than the length of stay described in a study from Southern Jordan where the average length of stay was 3.4 days.^45^ Al-Rubeaan^4^ found the mean duration for hospitalisation to be 6.56 ± 3.4 days. The longer duration of stay in our study could be multifactorial including lack of a dedicated team or unit caring for DKA patients and failure to institute protocols timeously because of the low index of suspicion in patients presenting with non-specific symptoms.

There was a mortality rate of 17.14% with the higher mortality occurring in patients with T2DM.^46,47,48^ This could be accounted for because of the older age and many more comorbidities compared to the T1DM group. Death rates from DKA in other studies were quite low compared to our study with the described range between 2% and 5%^15^ but these studies were conducted in HICs that are better resourced in terms of their infrastructure and have dedicated and experienced centres for DKA management. Death rates in African countries, which are mainly low- and middle-income countries (LMICs), remain high, as evidenced by mortality rate of 26% – 29% in countries such as Kenya, Tanzania and Ghana.^49^ Such variations in the mortality rates may reflect on the resources available in managing critically ill patients. The standard of care of such patients in HICs is very different compared to LMICs with subspecialty and intensive care unit facilities being more easily available in HICs.^50,51,52^ The mean age of those who died in this study was significantly higher compared to the survivors. This is consistent with what has been reported in other studies that were conducted in LMICs and HICs.^19,53,54^ In contrast, the study carried out at the University Teaching Hospital in Zambia reported that the mean age of patients who died following DKA hospitalisation was similar between the young and the old.^8^

Almost all the patients with measured glycated haemoglobin (HbA1c) were noted to have elevated levels of HbA1c, with most having levels above 10% indicating poor control which may have led them developing DKA. This is consistent with reports that patients with high HbA1c levels, especially greater than 10%, had higher risks of developing DKA^55,56^ and this is irrespective of diabetes type and duration.^28^ The resultant effect of elevated HbA1c levels is glucotoxicity to the beta cells which is associated with the development of unprovoked ketoacidosis.^57^

There was a significant difference in the markers of inflammatory conditions, that is, erythrocyte sedimentation rate (ESR) and C-reactive protein (CRP), between the T1DM and T2DM groups, the difference being higher in the latter group. This may be accounted for by a higher infection rate in the participants with T2DM. This finding has been previously documented where the CRP was reportedly elevated in DKA patients where infection was the precipitant.^24^ A higher infection rate in an older cohort with many more comorbidities may account for the significantly higher mortality rate in patients with T2DM.

## Limitations

This study had several limitations. Firstly, it was a retrospective review of records performed at one hospital that cares mainly for adults and excluded the paediatric population. There was no consistency in completion of clinical notes that may have contributed to an information bias. The study was conducted over a period of 10 months, which may have limited the ability to detect slight changes in the population.

The newly diagnosed diabetic patients were presumed to be T1DM by considering their phenotypic characteristics. Auto-antibodies and c-peptide levels were not measured to ascertain if they were objectively T1DM. The classification of diabetes was also based on the assumption that patients with certain phenotypical characteristics on injectables were T1DM and those on oral hypoglycaemic agents with or without injectables were T2DM. The blood ketone levels were not measured to confirm the diagnosis of DKA, and patients with acidosis and ketonuria may have been incorrectly classified as DKA. The glucose levels were only measured using the point of care glucometer, and in some cases, there was no confirmatory laboratory serum glucose. The individualised management of patients was not evaluated.

## Conclusion

Diabetic ketoacidosis is a common and often life-threatening complication of diabetes mellitus, especially in patients with T2DM with comorbid conditions. The overall mortality rate in this unit is worrying and is the indicator that there is room for improvement which needs to be addressed by the clinical managers in the hospital. High-risk patients presenting with DKA need to be rapidly assessed, and appropriate resuscitation involving a multidisciplinary team needs to be implemented. Early and appropriate critical care intervention may help reduce the mortality rate among the identified high-risk population. Community awareness of this serious complication is also needed, including routine screening for diabetes at primary health level coupled with at least a bi-annual monitoring of HbA1c levels.

Suggestions to improve patient’s outcome include encouraging younger patients to be involved in support groups, routine follow-up of patients that had presented in DKA, educating family members, clinicians and care givers about diabetes and the prevention of complications.
